# MS-BACL: enhancing metabolic stability prediction through bond graph augmentation and contrastive learning

**DOI:** 10.1093/bib/bbae127

**Published:** 2024-03-28

**Authors:** Tao Wang, Zhen Li, Linlin Zhuo, Yifan Chen, Xiangzheng Fu, Quan Zou

**Affiliations:** School of Data Science and Artificial Intelligence, Wenzhou University of Technology, 325000, Wenzhou, China; Institute of Computational Science and Technology, Guangzhou University, 510006, Guangzhou, China; School of Data Science and Artificial Intelligence, Wenzhou University of Technology, 325000, Wenzhou, China; College of Computer Science and Electronic Engineering, Hunan University, 410012, Changsha, China; College of Computer Science and Electronic Engineering, Hunan University, 410012, Changsha, China; Institute of Fundamental and Frontier Sciences, University of Electronic Science and Technology of China, 611730, Chengdu, China

**Keywords:** bond graph, contrastive learning, graph neural networks, metabolic stability, molecular structure

## Abstract

**Motivation:**

Accurately predicting molecular metabolic stability is of great significance to drug research and development, ensuring drug safety and effectiveness. Existing deep learning methods, especially graph neural networks, can reveal the molecular structure of drugs and thus efficiently predict the metabolic stability of molecules. However, most of these methods focus on the message passing between adjacent atoms in the molecular graph, ignoring the relationship between bonds. This makes it difficult for these methods to estimate accurate molecular representations, thereby being limited in molecular metabolic stability prediction tasks.

**Results:**

We propose the MS-BACL model based on bond graph augmentation technology and contrastive learning strategy, which can efficiently and reliably predict the metabolic stability of molecules. To our knowledge, this is the first time that bond-to-bond relationships in molecular graph structures have been considered in the task of metabolic stability prediction. We build a bond graph based on ‘atom-bond-atom’, and the model can simultaneously capture the information of atoms and bonds during the message propagation process. This enhances the model’s ability to reveal the internal structure of the molecule, thereby improving the structural representation of the molecule. Furthermore, we perform contrastive learning training based on the molecular graph and its bond graph to learn the final molecular representation. Multiple sets of experimental results on public datasets show that the proposed MS-BACL model outperforms the state-of-the-art model.

**Availability and Implementation:**

The code and data are publicly available at https://github.com/taowang11/MS.

## INTRODUCTION

Metabolic stability refers to the speed and degree of metabolism of compounds in organisms, and is an important observation indicator in drug discovery and clinical testing stages [1, 2]. The metabolic stability of a molecule largely determines its concentration and efficacy in the body, and profoundly affects the pharmacokinetic process [3, 4]. While certain molecules demonstrate potential as drug candidates, their poor metabolic stability in the body renders them unsuitable for current clinical use [5]. Accurately predicting the metabolic stability of molecules can provide a deep understanding of drug behavior in the body, thereby optimizing therapeutic dosage and ensuring efficacy [6] while controlling potential toxicity and risks resulting from drug interactions [7]. In the past few decades, human beings’ urgent needs for health have urgently required the development of a large number of symptomatic drugs. However, developing new drugs is often expensive and time-consuming, so efficient screening of candidate compounds from a large target space is critical [8]. Fortunately, predicting the metabolic stability of molecules can assist in screening the most promising compounds at an early stage, saving time and resources [9].

Traditionally, studying the metabolic stability of molecules has relied mainly on *in vitro* observations and assessments [10]. A common practice is to construct an *in vitro* model to simulate the metabolic environment in the body, and collect and evaluate observation data to provide guidance for subsequent *in vivo* research [11]. For example, in studies to determine the metabolic stability of candidate compounds, liver microsomes extracted from liver cells, including cytochrome P450 enzymes (a key class of drug-metabolizing enzymes), are used to assist in simulating the *in vivo* metabolic environment. Candidate compounds are then incubated with liver microsomes to observe and evaluate their metabolic rate [12]. However, observing and evaluating molecular metabolic stability in the laboratory relies on expensive experimental equipment, complex experimental design and a large amount of time. This has promoted the development of computational methods to predict molecular metabolic stability conveniently, quickly and accurately [13].

Recently, several relevant machine learning-based models have emerged to predict the metabolic stability of molecules. For instance, Perryman *et al*. [14] gathered data on mouse liver microsomal half-life in PubChem and proposed a model based on Bayesian theory to predict the metabolic stability of small molecules [15]. Rafael *et al*. created a tool to assess molecular metabolic stability, incorporating several machine learning algorithms such as random forests, support vector machines and naive Bayes [16]. Ryu *et al*. collected data on compounds in human liver microsomes and predicted the metabolic stability of these compounds based on a random forest model. In addition, Ryu *et al*. also evaluated the performance of various machine learning methods such as artificial neural networks, K-nearest neighbor algorithm and linear regression on the liver microsomal metabolic stability dataset. Machine learning methods can quickly predict the metabolic stability of molecules, but their performance is usually poor. The main reason is that the impact of the molecular structure of the compound on the metabolic stability is ignored.

Graph neural network (GNN) technology can efficiently understand structural and relational data, making it shine in topologically related biological research [17, 18]. This also includes inferring metabolic stability based on the topological structure of the molecule. For example, Renn *et al*. constructed a topological structure graph of molecules based on molecule smiles and used graph convolution network (GCN) technology to extract global features and local features. Subsequently, these two features are integrated to obtain the final molecular representation and thereby predict metabolic stability [19]. Du *et al*. constructed two views based on the molecular structure and used a graph contrastive learning strategy to train their topological features. In parallel, gated recurrent unit (GRU) and attention mechanisms are used to extract Smile-based features, which are integrated with topological features into the final molecular representation to predict the metabolic stability of the molecule [20].

Existing GNN models can efficiently predict the metabolic stability of molecules, but their performance is still limited by some inherent flaws. These GNN-based models focus more on the message propagation between nodes in the molecular graph while ignoring the relationship between bonds. As an important component of molecular graphs, bonds often play a key role in molecular properties such as metabolic stability. As a result, these models do not fully understand the structure of molecules, making it difficult to learn robust molecular representations. To this end, we propose a model named MS-BACL based on the bond graph augmentation and contrastive learning strategy, aiming to predict the metabolic stability of molecules efficiently and accurately. We construct a molecular graph based on molecular smiles, and construct a bond graph of the molecule based on ‘atom-bond-atom’ to reveal the structure of the molecule. In addition, we adopt a contrastive learning strategy to train molecular graphs and bond graphs to learn robust molecular representations. Multiple sets of experimental results on public datasets also prove that the proposed MS-BACL model can reliably predict molecular metabolic stability. In summary, our contributions are listed below:

1. We design the MS-BACL model based on the bond graph and contrastive learning strategy, which can reliably predict molecular metabolic stability.
2. This is the first time that the relationship between bonds in molecular graphs has been integrated into the molecular metabolic stability prediction task. The bond graphs are constructed based on ‘atom-bond-atom’, which supplements the topological structure information of the molecules. This enables the model to absorb both atomic and bond information during message propagation.
3. We use a contrastive learning strategy to train two views, molecular graph and bond graph, to learn robust molecular representations.
4. We construct multiple sets of experiments on public datasets to verify the effectiveness of the proposed MS-BACL model and key modules.

## METHOD

**Figure 1:**
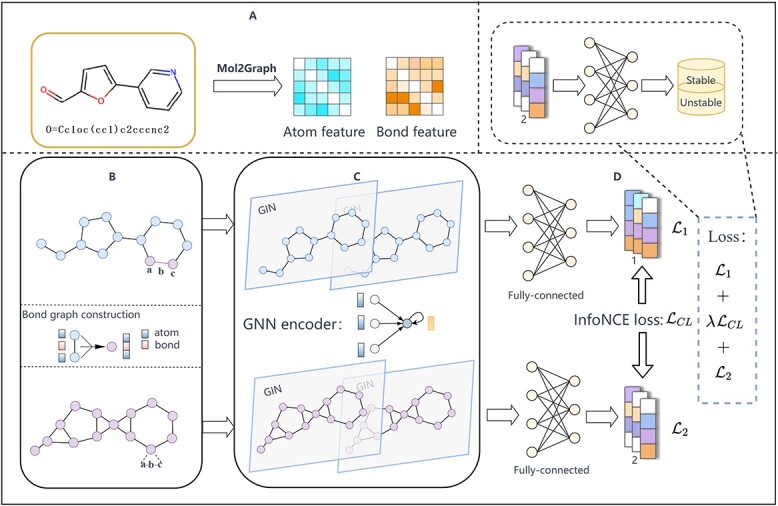
Architecture of MS-BACL model, which mainly contains four modules. (A) Constructing a molecular graph based on molecular smiles. (B) Define new nodes in the shape of ‘atom-bond-atom’ and build a bond graph. (C) Use GIN to extract features of molecular graphs and bond graphs. (D) Contrastive learning is used to train the model and predict the metabolic stability of molecules.

In this section, we propose the MS-BACL model based on bond graph augmentation technology and contrastive learning strategy to efficiently predict the metabolic stability of molecules. The MS-BACL model mainly includes the following modules, as shown in Figure 1. (A) First, we input the molecular SMILES into RDKit’s (https://pypi.org/project/rdkit/) conversion function to construct the molecular graph, thereby extracting features of atoms (nodes) and bonds (edges). (B) Then, we form a new node in the shape of ‘atom-bond-atom’ from the bond in the molecular graph and its two connected atoms, and build the bond graph of the molecule. In a bond graph, the model can take in both atom and bond information when performing aggregation and update operations. (C) Subsequently, we use graph isomorphism network (GIN) [21] to extract features of molecular graphs and molecular bond graphs, respectively. Following that, we perform global maximum and average pooling operations simultaneously on both graph representations before conducting a splicing operation to improve the node representation. (D) Finally, we calculate the classification loss on both the molecular graph and the molecular bond graph in parallel, incorporating the graph contrastive learning loss from both to learn the final molecular representation. Crucially, our predictions regarding the metabolic stability of molecules rely on the ultimate representation obtained from the bond graph. Next, we will introduce related technologies and principles in detail.

### Molecular bond graph

In graph theory, the edges in the original graph are regarded as nodes, and line graphs can be constructed accordingly [22]. The advantage of line graphs is that in the process of message passing, more consideration is given to the information on the edges and the relationship between the edges. In the molecular graphs, atoms and bonds are directly involved in the structure of the molecule, thus affecting the metabolic stability of the molecule. Inspired by the line graph, we will also consider the relationship between bonds and define new nodes in the shape of ‘atom-bond-atom’ to construct a bond graph. It is hoped that in the message passing, the information of atoms and bonds can be absorbed at the same time to enhance the molecular representation.

First, the smiles of the compound is taken as input and converted into a directed graph $G = (X, E, C)$. $X$ represents the set of all atom vectors in $G$, and the i-th atom vector is represented as $X_{i} \in X$. The atom vector includes the extracted atom symbol, total number of bonds, formal charge, number of bonded hydrogens, hybridization state, whether it is an aromatic system and the atomic mass. $E$ represents the set of all chemical bond vectors, and $E_{ij} \in E $ represents the bond vector from atom $i$ to $j$. The bond vector includes information such as the extracted bond type, whether it is conjugated and whether it is within a ring. $C$ represents the adjacency matrix of the molecular graph $G$, and $C_{ij} \in C $ represents whether there is a bond between atoms $i$ and $j$.

Then, we consider the relationship between chemical bonds, take ‘atom-bond-atom’ as a new node and construct a bond graph $G^{\prime} = (X^{\prime}, C^{\prime})$. In the bond graph $G^{\prime}$, $X^{\prime}$ represents all node vectors, and node $X_{ij}^{\prime}$ absorbs the eigenvectors of atoms $X_{i},\ X_{j} \in X$, and bonds $E_{ij} \in E $. $C^{\prime}$ represents the adjacency matrix of the bond graph $G^{\prime}$ and $C_{ik}^{\prime} \in C^{\prime}$ represents that the bonds $E_{ij},\ E_{jk} \in E $ exist at the same time and are adjacent to the atom $X_{j}$. Formally, $X^{\prime}$ and $C^{\prime}$ can be calculated by 


(1) \begin{align*} & X^{\prime}=\left\lbrace X_{ij}^{\prime}=X_{i} \Arrowvert E_{ij} \Arrowvert X_{j}, \ X_{i},\ X_{j} \in X and \ E_{ij} \in E \right\rbrace, \end{align*}

(2) \begin{align*} & C^{\prime}=\left\lbrace C_{ik}^{\prime}=1, C_{ij},\ C_{jk} \in C \right\rbrace, \end{align*}

where $\Arrowvert $ represents the concatenate operation. Finally, according to the above strategy, the molecular graph $G = (X, E, C)$ and the bond graph $G^{\prime} = (X^{\prime}, C^{\prime})$ are constructed based on molecular smiles.

### Molecular graph encoder

In the proposed MS-BACL model, we adopt the GIN model to extract the features of the molecular graph and bond graph. Molecular metabolic stability prediction can be considered as a graph classification task. Extracting local and global features of molecular graphs is very critical, and GIN is just qualified for this task. For the molecular graph $G = (X, E, C)$ and its corresponding molecular bond graph $G^{\prime} = (X^{\prime}, C^{\prime})$, the GIN encoder performs message aggregation and node updating based on node neighborhoods: 


(3) \begin{align*}& h_{i}^{k}=MLP\left( \left( 1+\epsilon^{k} \right) \cdot h_{i}^{k-1}+\sum_{j\in N(i)}h_{j}^{k-1} \right),\end{align*}

where $h_{i}^{k}$ represents the embedding of node $i$ in the $k$-th GIN layer, $\epsilon $ represents the weight parameter and $N(i)$ represents the neighbors of node $i$. Assuming the number of iterations is $K$, $h_{i}^{K}$ can effectively capture $K$-hop neighborhood information. Finally, global maximum and average pooling operations are performed on $h_{i}^{k}$, respectively, and the two vectors after the pooling operation are concatenated: 


(4) \begin{align*}& z_{i}^{k}=CONCAT(maxpool(h_{i}^{k}),meanpool(h_{i}^{k})).\end{align*}

Global max pooling emphasizes crucial features in molecular graphs. Global average pooling reduces noise impact on model performance and enhances its generalization capability. Integrating these two pooling strategies into the MS-BACL model seeks to optimize the emphasis on key features while enhancing generalization capacity.

### Graph contrastive learning strategy

Graph contrastive learning is an unsupervised learning strategy for graph data that aims to enhance the similarity between different views of graph data, thereby improving node representation. In the proposed MS-BACL model, we try to construct different views of the molecule (molecular graph and bond graph). The similarity score between two views of the same molecule is then increased to bring them closer to each other, thus providing complementary information. At the same time, the similarity scores between views of different molecules are reduced to distance them from each other, thereby discovering their differences.

Assuming that the total number of molecules in the training set is $M$, the molecular graph and bond graph are constructed based on smiles of each molecule. For a molecule $m$, $z_{m}$ and $z_{m}^{\prime}$ represent the extracted vectors of the molecular graph and its corresponding bond graph, respectively. And the InfoNCE function [23] is used to calculate the loss for contrastive learning training: 


(5) \begin{align*}& \mathcal{L}_{CL}=-\frac{1}{M}\sum_{m=1}^{M}log \frac{e^{z_{m} \cdot z_{m}^{\prime}/ \tau}}{\sum_{m^{\prime}=1}^{M} e^{z_{m} \cdot z_{m}^{\prime}/ \tau}},\end{align*}

where $\tau $ represents the temperature parameter, which was set to 0.5 in the experiment. In addition, if all molecules participate in contrastive learning training, it will consume a lot of time and space. Therefore, the contrastive learning process is usually completed within the sampling batch.

### Metabolic stability predictor

The optimization goal of the proposed MS-BACL model is to minimize both the classification and contrastive learning losses. We derived molecular representations from both molecular and bond graphs, utilizing each to predict the final metabolic stability score. The classification loss is computed using the BCE function: 


(6) \begin{align*} & \mathcal{L}_{1}=-\sum_{m=1}^{M}y_{m} \cdot log \sigma(\hat{y_{m}})+(1-y_{m}) \cdot log \sigma(1-\hat{y_{m}}); \end{align*}

(7) \begin{align*} & \mathcal{L}_{2}=-\sum_{m=1}^{M}y_{m} \cdot log \sigma(\hat{y_{m}}^{\prime})+(1-y_{m}) \cdot log \sigma(1-\hat{y_{m}}^{\prime}), \end{align*}

where $L_{1}$ represents the classification loss based on the molecular graph, $L_{2}$ represents the classification loss based on the bond graph, $M$ represents the number of molecules and $\sigma $ represents the sigmoid function. For the $m$-th molecule, where $\hat{y_{m}}$ represents the predicted score of metabolic stability based on the molecular graph, $\hat{y_{m}}^{\prime}$ represents the predicted score of metabolic stability based on the bond graph, and $y_{m}$ represents its true label. Integrating classification loss and contrastive learning loss: 


(8) \begin{align*}& \mathcal{L}=\mathcal{L}_{1}+\mathcal{L}_{2}+\lambda \mathcal{L}_{CL},\end{align*}

where $\lambda $ is an adjustable weight parameter.

During inference, metabolic stability is predicted using the representation derived from the extracted molecular bond graph. This differs subtly from the training procedure.

## EXPERIMENT RESULTS

### Datasets

In order to evaluate the performance of the proposed MS-BACL model, three datasets of molecular metabolic stability are mainly collected in the experiment. The first dataset, called HLM, concerns the metabolic stability of compounds on human liver microsomes and originated from Li *et al*.’s work [24]. There are currently no fixed and universally applicable unified criteria for defining metabolic stability. Referring to the study of Shah *et al*. [25], if the half-life of a molecule is greater than 30 min, it can be considered stable; otherwise, it is considered unstable. Accordingly, there are a total of 5876 molecules in the HLM dataset, including 3782 stable compounds and 2094 unstable compounds. The second is an external dataset [25], which includes 82 stable compounds and 29 unstable compounds. The third is a cross-species dataset, which is the rat microsome-related compounds we collected from the ChEMBL biological activity database (ID: 613694) [26], recorded as RLM. The RLM dataset contains a total of 499 molecules, including 208 stable compounds and 291 unstable compounds.

**Figure 2:**
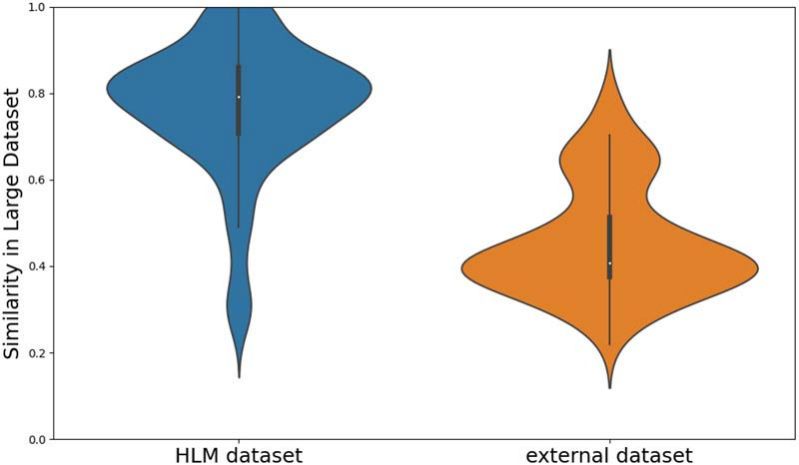
Distribution of HLM and external datasets.

To validate the model’s generalization capability, it was trained on the HLM dataset and subsequently assessed using an independent external dataset. To maintain experimental integrity and scientific rigor, we minimized the molecular structural similarity between the HLM dataset’s training set and the external dataset. Utilizing extended connectivity fingerprints and calculating the Tanimoto coefficient allowed for an efficient evaluation of structural similarity across numerous molecules. Figure 2 depicts the similarity distribution, with blue indicating the relationship between the training and test sets within the HLM dataset. The majority of similarity scores exceed 0.600, with an average of 0.766. Orange illustrates the similarity distribution between the external dataset and the HLM dataset’s training set, predominantly below 0.500 with an average of 0.456. Clearly, the similarity between the external dataset and the HLM dataset’s training set is markedly lower than that within the HLM dataset’s training and test sets. This confirms the reliability of the model’s performance evaluation on external datasets.

### Experimental setup

The proposed MS-BACL model is implemented based on Pytorch and PYG libraries. In the experiment, the number of layers of the GIN encoder is set to 2, the batchsize is 256, the number of training times is 300, the learning rate is 0.0005 and the optimizer is ADAM. In the predictor, the input layer has dimension 512, the hidden layer has dimension 256, the output layer has dimension 1 and the contrastive learning loss weight $\lambda $ is set to 0.3. In order to reduce the bias caused by the division of the dataset, we conduct a 10-fold cross-validation experiment, and the average value is used as the final result. In addition, we select four common indicators: $AUC$, $ACC$, $F1-Score$ and $MCC$ to evaluate the performance of the model.

### Performance comparison with other models

In this section, we compare the performance of the MS-BACL model and eight typical models. In experiments, we perform 10-fold cross-validation on each model to eliminate bias caused by randomness. To clarify the differences between the proposed MS-BACL model and the eight comparative models, we briefly introduce these models. Li *et al*. extracted molecular features based on GBDT and XGBoost integrated learning models, D-MPNN and GAT and other deep learning models to predict the metabolic stability of molecules [24]. The PredMS model uses random forest technology to extract important molecular descriptor features [14]. The MGCN model first constructs a molecular graph based on molecular smiles, and then uses GCN technology to learn molecular representation [19]. The AttentiveFP model uses GRU technology and graph attention mechanism to extract the representation of molecules, thereby accurately predicting the metabolic stability of molecules [27]. On this basis, the CMMS-GCL model further learned the molecular graph representation using a graph contrastive learning strategy, and finally integrated sequence representation and molecular graph representation to predict molecular metabolic stability [20].

We evaluate the performance of the MS-BACL model and eight other models on the HLM dataset. The results of 10-fold cross-validation are shown in Table 1. In general, methods based on deep GNN technology outperform methods based on ensemble learning strategies. PredMS is a model that uses an ensemble learning strategy, but it also relies on the chemical structure of the molecule to extract molecular representations. This illustrates that the chemical structure of the molecule plays a more critical role than the sequence when predicting the metabolic stability of the molecule. In addition, the proposed MS-BACL model is significantly better than all other models, and its $AUC$, $ACC$, $F1-Score$ and $MCC$ indicators are ahead of the suboptimal CMMS-GCL model 0.8%, 0.9%, 0.7% and 3.5%, respectively. This may be because the proposed MS-BACL model absorbs atom and bond information at the same time during message propagation, deeply revealing the mystery of the chemical structure of the molecules, and thereby more accurately predicting the metabolic stability. Unlike the CMMS-GCL model, which employs a graph contrastive learning strategy to improve molecular representation, it omits bond information during the message propagation process. Additionally, the AttentiveFP model utilizes a graph attention mechanism for extracting molecular representations, aiding in drug discovery efforts. While this model effectively captures complex atomic relationships, its performance lags behind MS-BACL due to a lack of consideration for bond interactions.

**Table 1:** Performance of all models on HLM dataset

Models	AUC	ACC	F1-Score	MCC
GBDT	0.815 ${\pm }$0.017	0.773${\pm }$0.013	0.830${\pm }$0.015	0.503${\pm }$0.025
XGBoost	0.844${\pm }$0.013	0.793${\pm }$0.022	0.846${\pm }$0.010	0.548${\pm }$0.026
D-MPNN	0.842${\pm }$0.017	0.792${\pm }$0.012	0.841${\pm }$0.013	0.541${\pm }$0.030
GAT	0.858${\pm }$0.016	0.782${\pm }$0.021	0.842${\pm }$0.015	0.533${\pm }$0.052
PredMS	0.854${\pm }$0.012	0.785${\pm }$0.021	0.843${\pm }$0.021	0.552${\pm }$0.104
MGCN	0.852${\pm }$0.019	0.784${\pm }$0.013	0.825${\pm }$0.018	0.544${\pm }$0.033
AttentiveFP	0.853${\pm }$0.015	0.793${\pm }$0.015	0.840${\pm }$0.013	0.564${\pm }$0.032
CMMS-GCL	0.865${\pm }$0.016	0.811${\pm }$0.015	0.856${\pm }$0.013	0.566${\pm }$0.040
MS-BACL	**0.873${\pm }$0.019**	**0.820${\pm }$0.023**	**0.863${\pm }$0.018**	**0.601${\pm }$0.053**

### Evaluation on external dataset

To verify the generalization ability of the proposed MS-BACL model, we evaluate the model trained in the HLM dataset on an external dataset, as shown in Table 2. The results show that the MS-BACL model outperforms existing leading models across all evaluated metrics. Notably, in terms of the $MCC$ metric, the MS-BACL model significantly surpasses the suboptimal CMMS-GCL model. This evidence underscores the MS-BACL model’s reliability in predictions and its adaptability to novel data.

**Table 2:** Performance of all models on external dataset

Models	AUC	ACC	F1-Score	MCC
GBDT	0.644${\pm }$0.046	0.740${\pm }$0.024	0.825${\pm }$0.013	0.155${\pm }$0.062
XGBoost	0.678${\pm }$0.018	0.732${\pm }$0.014	0.830${\pm }$0.011	0.150${\pm }$0.044
D-MPNN	0.766${\pm }$0.019	0.741${\pm }$0.013	0.852${\pm }$0.015	0.218${\pm }$0.038
GAT	0.814${\pm }$0.025	0.755${\pm }$0.052	0.825${\pm }$0.049	0.414${\pm }$0.081
PredMS	0.766${\pm }$0.014	0.756${\pm }$0.011	0.856${\pm }$0.006	0.231${\pm }$0.045
MGCN	0.830${\pm }$0.032	0.774${\pm }$0.033	0.845${\pm }$0.033	0.447${\pm }$0.064
AttentiveFP	0.816${\pm }$0.044	0.754${\pm }$0.034	0.814${\pm }$0.045	0.415${\pm }$0.067
CMMS-GCL	0.885${\pm }$0.015	0.836${\pm }$0.024	0.889${\pm }$0.017	0.569${\pm }$0.055
MS-BACL	**0.897${\pm }$0.017**	**0.842${\pm }$0.022**	**0.895${\pm }$0.016**	**0.588${\pm }$0.038**

### Ablation experiment

The proposed MS-BACL model mainly includes a contrastive learning module, a bond graph encoding module and a metabolic stability prediction module. In the experiments, we mainly explore the impact of the bond graph encoding module and the contrastive learning module on model performance. In addition, according to the analysis in Section 3.3, the metabolic stability of a molecule is greatly affected by the structure of the compound. Therefore, we study the impact of hydrogen atoms on model performance in order to reveal the key role of hydrogen atoms in the structure of compounds. In this study, ‘hydrogen atoms’ actually refer to non-framework hydrogen atoms.

Table 3 shows the results of the ablation experiments. In Table 3, ‘w/o GCL’ indicates removal of the graph contrastive learning module, followed by elimination of the original graph encoding module, leaving only the bond graph encoding module for molecular stability prediction. The ‘w/o BG’ setting implies that predictions of molecular metabolic stability are made using the original molecular graph, not the bond graph, while retaining both the graph contrastive learning and bond graph encoding modules throughout training. And ‘w/o H’ means that H atoms are deleted when constructing molecular and bond graphs, ‘w/o GCL & BG’ means that both the bond graph encoding and contrastive learning modules are removed and ‘w/o ALL’ means H atoms are deleted based on ‘w/o GCL & BG’. The results show that the performance of the model decreases after removing the contrastive learning or bond graph encoding module. At the same time, the contrastive learning and bond graph encoding modules are deleted, and only the GIN encoder was used to process molecular graphs, resulting in the worst performance of the model. In addition, we find that the performance of ‘w/o GCL & BG’ and ‘w/o H’ is almost the same, indicating that when the compound lacks key topological information, the use of bond graph encoding and contrastive learning modules can make up for it. This fully demonstrates the importance of bond graph encoding and contrastive learning modules to model performance.

**Table 3:** Results of ablation experiment

Models	AUC	ACC	F1-Score	MCC
w/o ALL	0.857${\pm }$0.023	0.795${\pm }$0.030	0.845${\pm }$0.018	0.552${\pm }$0.058
w/o H	0.864${\pm }$0.022	0.800${\pm }$0.030	0.848${\pm }$0.026	0.569${\pm }$0.052
w/o GCL & BG	0.860${\pm }$0.022	0.802${\pm }$0.027	0.850${\pm }$0.021	0.551${\pm }$0.055
w/o BG	0.866${\pm }$0.021	0.807${\pm }$0.025	0.852${\pm }$0.016	0.579${\pm }$0.048
w/o GCL	0.869${\pm }$0.022	0.811${\pm }$0.028	0.855${\pm }$0.023	0.586${\pm }$0.045
MS-BACL	**0.873${\pm }$0.019**	**0.820${\pm }$0.023**	**0.863${\pm }$0.018**	**0.601${\pm }$0.053**

In previous studies, when extracting the feature of the molecular structure or constructing a molecular graph, only heavy atoms were absorbed and the H atoms with the smallest molecular weight were ignored. We focused on exploring the impact of H atoms in the molecular structure on the metabolic stability of the model predicted molecules. The results of the ablation experiment show that the model performance decreases after deleting H atoms. This also proves that H atoms are very important in the molecular structure, enhancing the model to predict metabolic stability.

### Parameter analysis

In Equation 8, parameter $\lambda $ balances the classification loss with graph comparison learning loss. To identify the optimal $\lambda $ value, we designed experiments with $\lambda $ ranging from 0.1 to 0.9. Specifically, we split the HLM dataset into training and test sets at a 9:1 ratio, randomly designating one portion for testing and the rest for training. For each parameter experiment, we ensured consistency in the training and test sets, along with other parameters. Results depicted in Figure 4 reveal a stable performance of the model across $\lambda $ values [0.1, 0.9], with a slight decrease noted between [0.3, 0.9]. This indicates minimal impact of variations on model performance, facilitating the determination of $\lambda $ values for unknown datasets.

**Figure 3:**
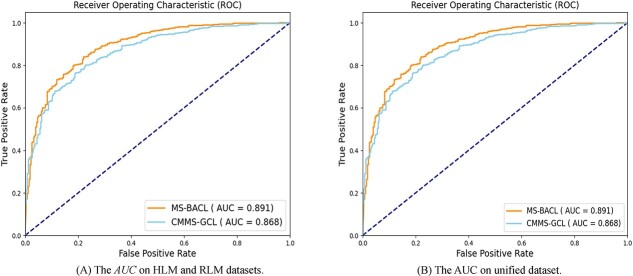
The $AUC$ performance of the MS-BACL and the suboptimal CMMS-GCL model on the cross-species metabolic stability dataset.

**Figure 4:**
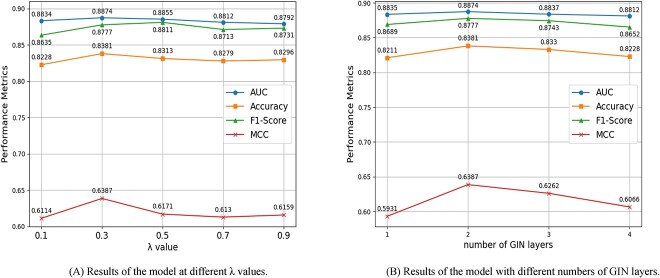
The performance of the MS-BACL across various parameter configurations.

Theoretically, an optimal number of layers enhances the GIN model’s ability to extract complex features, but excessive layers lead to an ‘over-smoothing’ issue. To assess the effect of the GIN model’s layer count on the MS-BACL model’s performance, we conducted parameter experiments to inform the optimal layer configuration. The experimental setup mirrors that of the experiment on parameter $\lambda $. Results presented in Figure 4 indicate that setting the GIN layers to 2 optimizes the model’s $AUC$, $ACC$, $F1-Score$ and $MCC$ metrics. Performance declines when exceeding two layers, demonstrating a negative correlation with the increase in GIN layers. This trend may lead the model toward ‘over-smoothing’. Thus, limiting the GIN model to fewer layers can circumvent this issue.

### Prediction of metabolic stability across species

In the early stages of drug development, the safety and efficacy of candidate compounds are often verified and evaluated on multiple biological models. In experiments, we collected metabolic stability data of compounds related to human liver microsomes and rat liver microsomes. We try to use the model trained based on the HLM dataset to evaluate the performance of the model on the RLM dataset. This is expected to help understand the similarities and differences in drug metabolism between humans and rats, thereby providing some new insights into the study of drug metabolism mechanisms.

Figure 3(a) presents the $AUC$ performance of the proposed MS-BACL and the suboptimal CMMS-GCL model on the cross-species metabolic stability dataset. The results show that the model trained on the HLM dataset has poor prediction performance on the RLM dataset. This indicates that the metabolic stability of compounds in human liver microsomes and rat liver microsomes is quite different. This difference highlights the complexity of predicting drug metabolism in different biological models and why multi-model drug testing is critical in the early stages of drug development. Therefore, cross-species prediction of metabolic stability helps to understand the behavior of drugs in different biological models and deeply explore and interpret the differences in metabolic mechanisms. Furthermore, the MS-BACL model trained on the HLM dataset performs better on the RLM dataset relative to the suboptimal CMMS-GCL model. This shows that the proposed MS-BACL model has better generalization ability and can explore the similarity of metabolic mechanisms in different species in cross-species metabolic stability prediction.

The extrapolation from model organisms, like rats, to humans is pivotal in drug development and biomedical research. Evaluating on a dataset that encompasses both model organisms and human data enhance the accuracy of drug effect predictions in humans and boost research productivity. Consequently, we integrated the HLM and RLM datasets to assess the model’s cross-species adaptability. The merged dataset encompasses both species, totaling 7332 samples with 4378 stable and 2954 unstable compounds. The training data comprised 1299 compounds from the RLM dataset and 5289 from the HLM dataset. The test set included 587 molecules from the HLM dataset and 157 from the RLM dataset. Figure 3(b) displays the $AUC$ metrics for MS-BACL and CMMS-GCL on the combined dataset. The experimental findings suggest that MS-BACL more precisely forecasts molecular metabolic stability across species datasets.

### Investigation of novel substructure

**Figure 5:**
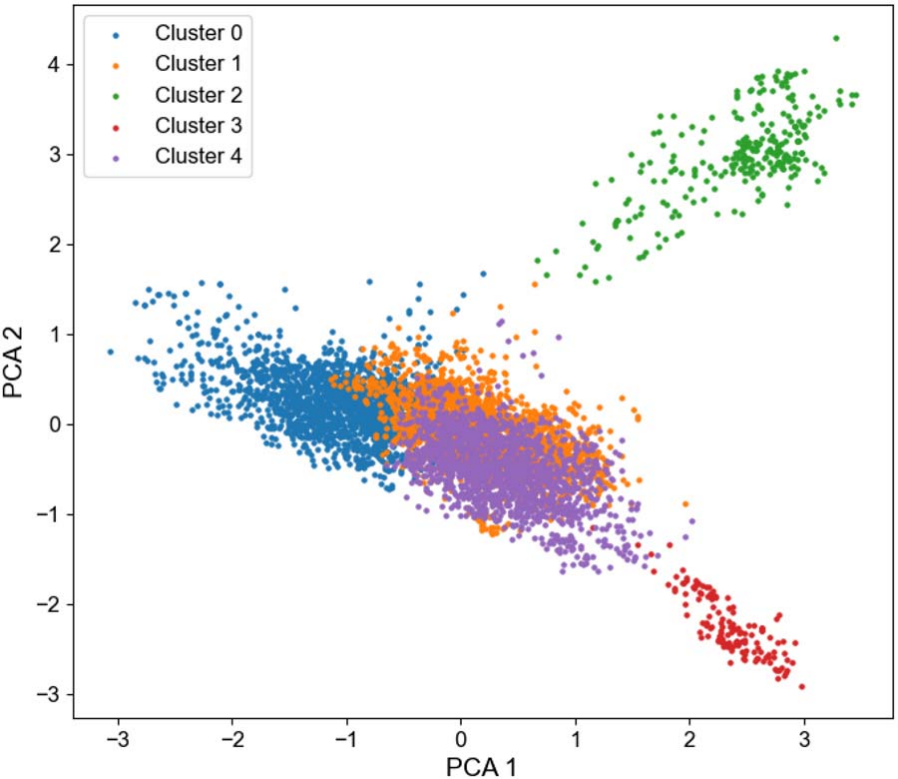
Distribution of five chemical structures in the HLM dataset.

We also evaluated the model’s performance in identifying novel molecular structures. ECFP fingerprinting was employed on the HLM dataset’s molecules, and the Tanimoto coefficient was calculated to ascertain molecular similarities. K-means clustering segregated the molecules into five distinct groups based on their structural attributes. Principal component analysis was utilized to reduce data dimensions for visual representation of the clustering outcomes. Five distinct clusters emerged, each with markedly different structures, as depicted in Figure 5.

**Figure 6:**
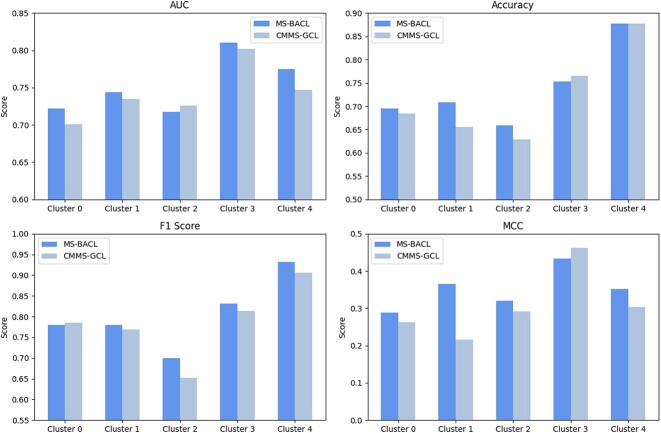
Performance of MS-BACL and suboptimal CMMS-GCL models in identifying novel and diverse chemical structures.

The ‘leave-one-out’ cross-validation approach involves segmenting the dataset into five clusters via K-means method, with one cluster designated as the test set in each iteration, and the other clusters serving as the training set. Rotating leave-one-out cross-validation across five clusters assessed each model’s capability to recognize novel structural molecules. Results, presented in Figure 6, reveal that the MS-BACL model’s $AUC$, $ACC$, $F1-score$ and $MCC$ metrics significantly surpass those of contemporary leading models. This corroborates the MS-BACL model’s effectiveness in identifying novel molecular structures and its adaptability to diverse structural variations.

Figure 5 reveals that Cluster2 exhibits a relatively dispersed node distribution. This dispersion likely stems from the low structural similarity among the cluster’s compounds, leading to significant property variances. Consequently, Figure 6 shows that both the MS-BACL and the CMMS-GCL models exhibit reduced predictive accuracy. In contrast to Cluster2, Cluster3 displays a tighter node distribution, indicating higher structural similarity among its compounds and minimal property differences. As a result, both the MS-BACL and CMMS-GCL models demonstrate enhanced predictive performance. Additionally, within Cluster2, the MS-BACL model slightly underperforms the CMMS-GCL model in $AUC$ index, possibly due to the substantial chemical structure dissimilarity among samples, influenced by random factors. In Cluster3, the MS-BACL model falls marginally behind the CMMS-GCL model in $ACC$ and $MCC$ metrics, a discrepancy that could be attributed to the limited sample size and random factors. Overall, the MS-BACL model outperforms the CMMS-GCL model in both Cluster2 and Cluster3.

### Effect of substructure on metabolic stability

In this section, we reveal in depth the dependencies of model effectiveness and explore key atoms or substructures that influence metabolic stability. This not only improves the interpretability of model prediction results, but also provides valuable guidance for compound design and optimization. Molecular substructure analysis is performed on the testset of the HLM dataset, which included 383 positive samples and 202 negative samples. We construct a bond graph from molecular SMILES and estimate the Shapley values of its nodes using a method akin to EdgeSHAPer [28]. These nodes encapsulate the properties of chemical bonds and adjacent atoms, ensuring that the derived Shapley values are imbued with extensive chemical information. Mapping these Shapley values to their respective locations within the molecule’s original structure allows for a more precise analysis of each functional group or chemical bond’s effect on the molecule’s predicted metabolic stability. Generally speaking, unstable functional groups have a greater impact on the metabolic stability of compounds. Therefore, we count the frequency of occurrence of functional groups or bonds that have a negative effect on the metabolic stability of compounds.

**Figure 7:**
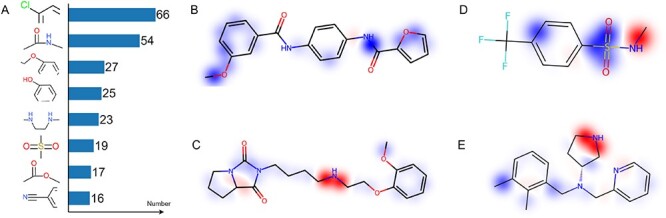
The frequency of occurrence of functional groups or bonds that have a negative effect on the metabolic stability of compounds.

We focus on bonds with Shapley values less than -0.4, and screen out the top eight functional groups containing these bonds that have a greater impact on the model’s predicted metabolic instability, as shown in Figure 7.A. Figure 7.B and D shows the structure of the metabolically unstable compounds, and Figure 7.C and E shows the structure of the metabolically stable compounds. The blue part indicates a negative impact on metabolic stability, the red part indicates a positive impact on metabolic stability and the depth of the color indicates the degree of impact. In Figure 7.B, it can be found that the amide functional group and the ether bond connecting the benzene ring enhance the metabolic instability of the compound. Figure 7.D indicates that secondary amines contribute to the compound’s stability, while the sulfonyl functional group induces metabolic instability, resulting in the compound’s overall instability during metabolism. In Figure 7.C and E, there are no functional groups that significantly enhance metabolic instability. On the contrary, the secondary amine structure enhances the metabolic stability of the compound. The results show that the proposed MS-BACL model can identify specific structures to predict metabolic stability, and can reveal the impact of chemical structures on metabolic stability. This optimizes the drug design task at an early screening stage by avoiding the generation of potential structures that are unstable or prone to breakdown *in vivo*.

## CONCLUSION

In this study, we first investigated related methods for predicting the metabolic stability of molecules and pointed out some limitations of these methods. For example, machine learning-based methods only extract features such as molecular sequences, but do not consider the chemical structure of the molecules. Deep learning, especially GNN-related methods, can efficiently predict molecular stability relying on the chemical structure of the molecule, but only focus on the message propagation on atoms and ignore the information of chemical bonds. To this end, we propose the MS-BACL model based on bond graph augmentation and contrastive learning strategies, aiming to reliably predict the metabolic stability of compounds. The proposed MS-BACL model constructs a bond graph that captures the relationship between bonds. This enables the MS-BACL model to absorb both atomic and chemical bond information in message passing, thus enhancing the structural representation of molecules. In addition, we conduct contrastive training based on molecular graphs and their bond graphs to learn robust molecular representations and improve model performance.

We construct multiple sets of comparison and ablation experiments on HLM, and external datasets to verify the performance of the proposed MS-BACL model and the role of its key modules. Experiments on human and rat metabolism datasets can understand the similarities and differences in drug metabolism of different species to a certain extent. We count the frequency of functional groups that lead to a decrease in metabolic stability and analyzed the impact of key substructures of molecules on metabolic stability. In addition, we also explore the impact of small molecular weight H atoms on the chemical structure of the molecule. These results and analyses prove that the MS-BACL model can indeed reliably predict the metabolic stability of molecules, and are also expected to provide valuable reference for drug design and optimization.

The high efficiency of the proposed MS-BACL model particularly relies on the bond graph encoding module, which can simultaneously absorb atom and chemical bond information during the message propagation process. This bond graph strategy is pluggable and can be easily embedded into other GNN-related models. It can be thus widely used to solve graph-related bioinformatics problems, especially to understand and reveal information about the chemical structure of molecules. Nonetheless, the model presents certain limitations. First, the model solely extracts features from molecular chemical structures, neglecting multi-source data like sequences, images and text descriptions. Secondly, training exclusively on a specific dataset hampers the acquisition of generalized molecular representation, leading to limited generalization capabilities. For future work, we intend to incorporate multi-source data to refine molecular representation and employ pre-training or large language models to learn general knowledge of moleculars and enhance the model’s generalization capacity.

**Key Points**

- The designed MS-BACL model demonstrates a reliable capability in predicting molecular metabolic stability.
- A novel ‘atom-bond-atom’ based molecular bond graph enhances molecule topological data, facilitating atom and bond information absorption during model message propagation.
- A contrastive learning strategy is adeptly utilized to train molecular and bond graphs, effectively honing robust molecular representations.

## Data Availability

Our code and data are accessible at https://github.com/taowang11/MS.

## Author Biographies

**Tao Wang** pursued his studies at the Wenzhou University of Technology under the guidance of Linlin Zhuo.

**Zhen Li** received her PhD in Ecology from Sun Yat-sen University. She is currently a postdoctoral research fellow in Institute of Computing Science and Technology at Guangzhou University, China, advised by Prof. Wenbin Liu. Her research interests focus on bioinformatics, machine learning, and mathematical algorithm.

**Linlin Zhuo**, an associate professor at the Wenzhou University of Technology, focuses his research on bioinformatics.

**Yifan Chen** received the PhD degrees in the College of Computer Science and Electronic Engineering from Hunan University, Changsha, China.

**Xiangzheng Fu** received the MS and PhD degrees in the College of Computer Science and Electronic Engineering from Hunan University, Changsha, China, in 2014 and 2019, respectively. His current research interests include Bioinformatics, Drug discovery, and Machine learning.

**Quan Zou** is a Professor of Institute of Fundamental and Frontier Sciences, University of Electronic Science and Technology of China. His research is in the areas of bioinformatics, machine learning and parallel computing. Now he is putting the focus on protein classification, genome assembly, annotation and functional analysis from the next generation sequencing data with parallel computing methods.
